# Beyond Thirty Days: Risk, Reintervention, and Recovery in Contemporary Cardiovascular Surgery

**DOI:** 10.3390/jcdd13090461

**Published:** 2026-09-14

**Authors:** Vasiliki Androutsopoulou, Prokopis-Andreas Zotos, Dimitrios E. Magouliotis

**Affiliations:** 1 Department of Cardiothoracic Surgery, University Hospital of Larissa, 41110 Larissa, Greece; androutsopoulouvasiliki@uth.gr (V.A.); zotospro@hotmail.com (P.-A.Z.); 2 Department of Cardiac Surgery Research, Lankenau Institute for Medical Research, Main Line Health, Wynnewood, PA 19096, USA

## 1. Introduction

Cardiovascular surgery measures itself in operative mortality. The instruments that the specialty built reflect that choice: risk scores calibrated to in-hospital death [1], trials powered for thirty-day endpoints, and quality metrics anchored to the index admission. That framework served well when patients were generally old and the operation was the last one they would undergo; it serves poorly now. A pooled analysis of six randomized trials enrolling 5341 lower-risk patients found a 20% reduction in the hazard of death at five years with transcatheter compared with surgical aortic valve replacement, and the authors were explicit in stating that most patients had not yet reached five-year follow-up and that data in younger patients remain the outstanding requirement [2]. Patients in their sixties now receive prostheses that will need to be revised, and the choice of first procedure constrains every subsequent one [3]. Coronary devices are engineered to disappear on a schedule. Dialysis-dependent patients survive long enough for conduit selection to matter, or not to.

The decisions have acquired a horizon measured in decades. The tools used to make them still stop at day 30.

This Special Issue, “Contemporary Controversies and Evolving Paradigms in Cardiovascular Surgery,” assembles five contributions published between July and December 2025. They address coronary revascularization in dialysis-dependent patients (Contribution 1), surgical explantation of transcatheter aortic valves (Contribution 2), the management of infective endocarditis after transcatheter aortic valve replacement (Contribution 3), bioresorbable coronary scaffolds (Contribution 4), and fast-track extubation after cardiac surgery (Contribution 5). They were solicited independently and they converge. Each describes a decision made under one time horizon and judged under another.

## 2. When the Risk Model Outlives Its Calibration

Deniz and colleagues compared 97 dialysis-dependent patients undergoing coronary artery bypass grafting with 488 non-dialysis-dependent controls at a single institution (Contribution 1). Perioperative mortality was 10.3% versus 3.1%, and five-year survival was 40.8% versus 82.1%. The survival gap was expected; what happened to the risk score was not. EuroSCORE II discriminated acceptably for perioperative death among dialysis patients, with an area under the curve of 0.783, and fell to 0.669 at five years. In the non-dialysis cohort, the same score held at 0.750 over the same interval. The model did not simply lose accuracy with time; it lost accuracy fastest in the population where the long-term question is hardest to answer.

This is a specific instance of a general result. Barili and colleagues linked 10,033 consecutive cardiac surgical patients to a national tax register and showed both discrimination and calibration of EuroSCORE II decay beyond thirty days, while its component variables remain independent predictors of late death [4]. EuroSCORE II was derived on 22,381 patients with in-hospital mortality as its primary outcome, and it performs as designed [1]. The fault does not lie in the score; it lies in using an instrument built for one horizon to settle questions posed at another.

The same contribution reports a negative finding worth preserving. Arterial grafting improved survival in the non-dialysis cohort but conferred no measurable benefit in dialysis patients (*p* = 0.597). Median survival in the dialysis cohort was 3.2 years, short of the interval over which saphenous vein graft patency begins to fail. When the patient does not outlive the conduit, conduit choice stops being the operative question. Long-term evidence still favors surgical revascularization in dialysis-dependent patients, with an odds ratio for late death of 0.81 against percutaneous intervention across 18 studies and 69,456 patients [5], and guidelines continue to recommend it [6]. Reconciling that recommendation with a five-year survival of 40.8% requires a risk model that reports the horizon the patient actually faces.

## 3. Devices That Have to Come Out

Androutsopoulou and colleagues review the surgical explantation of transcatheter aortic valves (Contribution 2). The operation is now among the fastest-growing procedures in cardiac surgery, and until recently it had no dedicated risk instrument. Reported thirty-day mortality ranges from 15% to 20%, and observed-to-expected mortality ratios run from 1.5 to 3.5. Every established score underestimates it.

The registry data are consistent and unfavorable. In the EXPLANT-TAVR international registry, in-hospital, thirty-day and one-year mortality after explantation were 11.9%, 13.1% and 28.5% [7]. Among 391 patients, the median interval from implantation to explantation was 13.3 months, endocarditis accounted for 36.0% of self-expanding and 55.4% of balloon-expandable valve failures, and concomitant cardiac procedures were required in 57.8% [8]. In a subsequent analysis of 372 patients, one-year mortality was 33.8%, and those explanted for endocarditis had higher stroke rates at thirty days (8.6% versus 2.9%) and at one year (16.2% versus 5.2%) [9]. A single-center series of 66 reoperations after transcatheter replacement found that low- and intermediate-risk patients had become the predominant group requiring them [10].

The field is responding. The Heart Valve Collaboratory has published a consensus operative technique for explantation [11]. The Society of Thoracic Surgeons has progressively extended its model portfolio, adding risk models for multiple valve operations [12] and, most recently, for aortic valve replacement after transcatheter replacement, alongside linkage to the National Death Index for longitudinal survival reporting [13]. Both developments are necessary. Both arrived after the procedures they describe became common, which is the recurring pattern across this Special Issue.

The conclusion Androutsopoulou and colleagues reach follows from the numbers: the risk of explantation belongs in the conversation at the index procedure, not at reoperation. That conversation currently takes place between a patient and an interventional team, often years before a surgeon is involved.

## 4. Guideline-Indicated Surgery That Does Not Happen

Magouliotis and colleagues pooled three studies comprising 1557 patients with infective endocarditis after transcatheter aortic valve replacement, of whom 155 (10.0%) underwent surgery (Contribution 3). Thirty-day mortality was 9.7% in the surgical group and 8.4% in the medical group. The pooled odds ratio for one-year survival was 1.91 (95% CI 0.36 to 10.22), with an I-squared of 88%. The pooled estimate is uninformative; the heterogeneity is the finding. One single-center series reported one-year survival of 96% with surgery against 51% with medical therapy, and the largest multicenter registry found no difference.

Two readings are available. Surgery may work where it is performed early and by experienced teams. Alternatively, a center operating on more than half of its cases is selecting different patients from a center operating on 2.5%. Both are probably true, and the available data cannot separate them. The denominator, however, is not in dispute. The incidence of endocarditis after transcatheter replacement has remained stable as the procedure expanded, at rates comparable to those after surgical replacement, and the prognosis remains poor [14]. In the Infective Endocarditis after TAVI International Registry, 54.4% of 579 patients had a clinical indication for surgery and fewer than one in five received it [15]. In a treatment-effect analysis of 584 patients from the same registry, 111 (19%) were managed surgically, and after adjustment for selection and immortal time bias, surgery was not associated with lower in-hospital or one-year mortality [16].

That last result should be read with precision. It states that surgery, applied to the patients currently selected at the time they are currently referred, does not improve survival. It does not state that earlier referral would not. The strongest evidence bearing on that question is organizational rather than technical: implementation of a multidisciplinary endocarditis team was followed by a fall in in-hospital mortality from 29.4% to 7.1% among patients with definite endocarditis and a surgical indication [17]. The operation did not change; the structure around it did.

## 5. Devices Designed to Disappear

Bourazana and colleagues trace bioresorbable coronary scaffolds from concept to clinical maturity (Contribution 4). This literature is the cleanest available natural experiment in time-dependent risk. In AIDA, which randomized 1845 patients in routine practice, two-year target-vessel failure was equivalent between scaffold and metallic stent (11.7% versus 10.7%), while definite or probable device thrombosis was 3.5% versus 0.9% [18]. In ABSORB IV, with improved implantation technique in 2604 patients, five-year target lesion failure was 17.5% versus 14.5%; the excess risk was confined to the first three years, the period ending with complete resorption, and event rates were similar between three and five years [19].

The hazard had a start date and an end date. Pathological work has already established why, showing that the vascular healing response to a degrading polymer differs from the response to a permanent metallic platform [20]. Magnesium-based scaffolds have since narrowed the early penalty, with a 24-month target lesion failure rate of 6.8% and definite or probable scaffold thrombosis of 0.8% among 2066 patients [21].

The transferable lesson is not about scaffolds. When a device changes its mechanical properties over time, a single hazard ratio conceals more than it reports, and a trial that closes before the device finishes changing has answered a different question from the one that was asked.

## 6. The Window That Remains Fully Modifiable

Christophides and colleagues review fast-track extubation after cardiac surgery (Contribution 5). Twenty-eight randomized trials enrolling 4438 patients established that low-dose opioid anesthesia and time-directed extubation protocols carry mortality and major complication rates equivalent to conventional care, while shortening time to extubation and intensive care stay without shortening total hospital stay [22]. The predictors of failure are intraoperative and largely modifiable: cardiopulmonary bypass duration, inotropic requirement, and transfusion. Bypass time beyond 120 min doubled the risk of delayed extubation in one derivation cohort.

Two boundaries deserve mention. Overnight extubation, long avoided, was not associated with increased mortality or reintubation among 39,812 patients in a multicenter database, although those extubated between midnight and 06:00 showed higher composite morbidity [23]. And fast-track extubation is an element of a pathway rather than a pathway in itself; both the ERAS Cardiac recommendations and the joint ERAS and STS consensus place it inside a protocol that begins before the incision [24,25].

This contribution functions as a counterweight. Among four papers concerned with consequences that arrive years later, it describes the interval in which the causal chain is short and the levers are real.

## 7. Where the Work Should Go

Four priorities follow from these contributions.

First, risk models need declared horizons. A score reporting in-hospital mortality answers the surgeon’s question, not the patient’s. Recalibrating existing variables to one-year and five-year endpoints is tractable and requires no new data collection [4], and registry linkage to national death indices makes longitudinal reporting feasible at scale [13]. Machine learning improves discrimination for operative mortality, with a pooled C-statistic of 0.88 against 0.81 for logistic regression across 15 studies, although the clinical magnitude of that gain remains uncertain [26]. Extending the outcome window is likely to matter more than changing the algorithm. Population-specific models are also required where the general model demonstrably fails, and dialysis dependence is the clearest example (Contribution 1).

Second, the index decision should be documented as a lifetime decision. Guidelines already frame valve intervention this way [27,28]. Practice does not consistently follow. A 65-year-old receiving a transcatheter valve should have the reintervention pathway, including explantation and its measured mortality, recorded as part of the informed consent (Contribution 2).

Third, referral structure should be evaluated as an intervention in its own right. The endocarditis team data indicate that the largest available gains in endocarditis after transcatheter replacement are organizational [17]. Prospective evaluation of structured triage pathways, with surgical consultation as a default rather than a rescue, is overdue (Contribution 3).

Fourth, follow-up should outlast the device. This applies to scaffolds that resorb at three years (Contribution 4), to transcatheter valves whose failure declares itself at a median of 13 months (Contribution 2), and to venous conduits whose patency curve crosses the patient’s survival curve somewhere in the fourth year (Contribution 1).

## 8. Conclusions

The five contributions to this Special Issue share a structure. Deniz and colleagues show a validated score losing discrimination inside five years. Androutsopoulou and colleagues describe an operation performed without a risk model. Magouliotis and colleagues quantify guideline-indicated surgery withheld from 90% of eligible patients. Bourazana and colleagues trace a device whose entire risk profile is a function of time since implantation, and Christophides and colleagues define the interval over which outcomes remain fully modifiable. Contemporary controversy in cardiovascular surgery is less about which operation to perform than about when the result should be counted. Extending the measurement window is not a methodological refinement; it is the substance of the argument.

## Data Availability

No new data were created or analyzed in this study. Data sharing is not applicable to this article.

## References

[1] Nashef S.A.M., Roques F., Sharples L.D., Nilsson J., Smith C., Goldstone A.R., Lockowandt U. (2012). EuroSCORE II. Eur. J. Cardiothorac. Surg..

[2] Reddy R.K., Howard J.P., Mack M.J., Reardon M.J., Jørgensen T.H., Hørsted Thyregod H.G., Toff W.D., Van Mieghem N.M., Vora A.N., Makkar R.R. (2025). Transcatheter vs Surgical Aortic Valve Replacement in Lower-Risk Patients: An Updated Meta-Analysis of Randomized Controlled Trials. J. Am. Coll. Cardiol..

[3] Jubran A., Patel R.V., Sathananthan J., Wijeysundera H.C. (2024). Lifetime Management of Patients With Severe Aortic Stenosis in the Era of Transcatheter Aortic Valve Replacement. Can. J. Cardiol..

[4] Barili F., Pacini D., D’Ovidio M., Dang N.C., Alamanni F., Di Bartolomeo R., Grossi C., Davoli M., Fusco D., Parolari A. (2016). The Impact of EuroSCORE II Risk Factors on Prediction of Long-Term Mortality. Ann. Thorac. Surg..

[5] Bundhun P.K., Bhurtu A., Chen M.-H. (2016). Impact of Coronary Artery Bypass Surgery and Percutaneous Coronary Intervention on Mortality in Patients with Chronic Kidney Disease and on Dialysis: A Systematic Review and Meta-Analysis. Medicine.

[6] Lawton J.S., Tamis-Holland J.E., Bangalore S., Bates E.R., Beckie T.M., Bischoff J.M., Bittl J.A., Cohen M.G., DiMaio J.M., Don C.W. (2022). 2021 ACC/AHA/SCAI Guideline for Coronary Artery Revascularization. Circulation.

[7] Bapat V.N., Zaid S., Fukuhara S., Saha S., Vitanova K., Kiefer P., Squiers J.J., Voisine P., Pirelli L., von Ballmoos M.W. (2021). Surgical Explantation After TAVR Failure: Mid-Term Outcomes From the EXPLANT-TAVR International Registry. JACC Cardiovasc. Interv..

[8] Zaid S., Kleiman N.S., Goel S.S., Szerlip M.I., Mack M.J., Marin-Cuartas M., Mohammadi S., Nazif T.M., Unbehaun A., Andreas M. (2024). Impact of Transcatheter Heart Valve Type on Outcomes of Surgical Explantation After Failed Transcatheter Aortic Valve Replacement: The EXPLANT-TAVR International Registry. EuroIntervention.

[9] Marin-Cuartas M., Tang G.H.L., Kiefer P., Fukuhara S., Lange R., Harrington K.B., Saha S., Hagl C., Kleiman N.S., Goel S.S. (2024). Transcatheter Heart Valve Explant with Infective Endocarditis-Associated Prosthesis Failure and Outcomes: The EXPLANT-TAVR International Registry. Eur. Heart J..

[10] Fukuhara S., Kim K.M., Yang B., Romano M., Ailawadi G., Patel H.J., Deeb G.M. (2024). Reoperation Following Transcatheter Aortic Valve Replacement: Insights from 10 Years’ Experience. J. Thorac. Cardiovasc. Surg..

[11] Kaneko T., Bapat V.N., Alakhtar A.M., Zaid S., George I., Grubb K.J., Harrington K., Pirelli L., Atkins M., Desai N.D. (2025). Transcatheter Heart Valve Explantation for Transcatheter Aortic Valve Replacement Failure: A Heart Valve Collaboratory Expert Consensus Document on Operative Techniques. J. Thorac. Cardiovasc. Surg..

[12] Jacobs J.P., Shahian D.M., Badhwar V., Thibault D.P., Thourani V.H., Rankin J.S., Kurlansky P.A., Bowdish M.E., Cleveland J.C., Furnary A.P. (2022). The Society of Thoracic Surgeons 2021 Adult Cardiac Surgery Risk Models for Multiple Valve Operations. Ann. Thorac. Surg..

[13] Iribarne A., Zwischenberger B., Mehaffey J.H., Kaneko T., Wyler von Ballmoos M.C., Jacobs J.P., Krohn C., Habib R.H., Parsons N., Badhwar V. (2025). The Society of Thoracic Surgeons Adult Cardiac Surgery Database: 2024 Update on National Trends and Outcomes. Ann. Thorac. Surg..

[14] Del Val D., Panagides V., Mestres C.A., Miró J.M., Rodés-Cabau J. (2023). Infective Endocarditis After Transcatheter Aortic Valve Replacement: JACC State-of-the-Art Review. J. Am. Coll. Cardiol..

[15] Panagides V., Abdel-Wahab M., Mangner N., Durand E., Ihlemann N., Urena M., Pellegrini C., Giannini F., Scislo P., Huczek Z. (2022). Sex Differences in Infective Endocarditis After Transcatheter Aortic Valve Replacement. Can. J. Cardiol..

[16] Mangner N., del Val D., Abdel-Wahab M., Crusius L., Durand E., Ihlemann N., Urena M., Pellegrini C., Giannini F., Gasior T. (2022). Surgical Treatment of Patients With Infective Endocarditis After Transcatheter Aortic Valve Implantation. J. Am. Coll. Cardiol..

[17] El-Dalati S., Cronin D., Riddell J., Shea M., Weinberg R.L., Washer L., Stoneman E., Perry D.A., Bradley S., Burke J. (2022). The Clinical Impact of Implementation of a Multidisciplinary Endocarditis Team. Ann. Thorac. Surg..

[18] Wykrzykowska J.J., Kraak R.P., Hofma S.H., van der Schaaf R.J., Arkenbout E.K., IJsselmuiden A.J., Elias J., van Dongen I.M., Tijssen R.Y.G., Koch K.T. (2017). Bioresorbable Scaffolds versus Metallic Stents in Routine PCI. N. Engl. J. Med..

[19] Stone G.W., Kereiakes D.J., Gori T., Metzger D.C., Stein B., Erickson M., Torzewski J., Kabour A., Piegari G., Cavendish J. (2023). 5-Year Outcomes After Bioresorbable Coronary Scaffolds Implanted With Improved Technique. J. Am. Coll. Cardiol..

[20] Jinnouchi H., Torii S., Sakamoto A., Kolodgie F.D., Virmani R., Finn A.V. (2019). Fully Bioresorbable Vascular Scaffolds: Lessons Learned and Future Directions. Nat. Rev. Cardiol..

[21] Wlodarczak A., Montorsi P., Torzewski J., Bennett J., Starmer G., Buck T., Haude M., Moccetti M., Wiemer M., Lee M.K.-Y. (2023). One- and Two-Year Clinical Outcomes of Treatment with Resorbable Magnesium Scaffolds for Coronary Artery Disease: The Prospective, International, Multicentre BIOSOLVE-IV Registry. EuroIntervention.

[22] Wong W.-T., Lai V.K.W., Chee Y.E., Lee A. (2016). Fast-Track Cardiac Care for Adult Cardiac Surgical Patients. Cochrane Database Syst. Rev..

[23] Krebs E.D., Hawkins R.B., Mehaffey J.H., Fonner C.E., Speir A.M., Quader M.A., Rich J.B., Yarboro L.T., Teman N.R., Ailawadi G. (2019). Is Routine Extubation Overnight Safe in Cardiac Surgery Patients?. J. Thorac. Cardiovasc. Surg..

[24] Engelman D.T., Ben Ali W., Williams J.B., Perrault L.P., Reddy V.S., Arora R.C., Roselli E.E., Khoynezhad A., Gerdisch M., Levy J.H. (2019). Guidelines for Perioperative Care in Cardiac Surgery: Enhanced Recovery After Surgery Society Recommendations. JAMA Surg..

[25] Grant M.C., Crisafi C., Alvarez A., Arora R.C., Brindle M.E., Chatterjee S., Ender J., Fletcher N., Gregory A.J., Gunaydin S. (2024). Perioperative Care in Cardiac Surgery: A Joint Consensus Statement by the Enhanced Recovery After Surgery (ERAS) Cardiac Society, ERAS International Society, and The Society of Thoracic Surgeons (STS). Ann. Thorac. Surg..

[26] Benedetto U., Dimagli A., Sinha S., Cocomello L., Gibbison B., Caputo M., Gaunt T., Lyon M., Holmes C., Angelini G.D. (2022). Machine Learning Improves Mortality Risk Prediction After Cardiac Surgery: Systematic Review and Meta-Analysis. J. Thorac. Cardiovasc. Surg..

[27] Otto C.M., Nishimura R.A., Bonow R.O., Carabello B.A., Erwin J.P., Gentile F., Jneid H., Krieger E.V., Mack M., McLeod C. (2021). 2020 ACC/AHA Guideline for the Management of Patients With Valvular Heart Disease. Circulation.

[28] Vahanian A., Beyersdorf F., Praz F., Milojevic M., Baldus S., Bauersachs J., Capodanno D., Conradi L., De Bonis M., De Paulis R. (2022). 2021 ESC/EACTS Guidelines for the Management of Valvular Heart Disease. Eur. Heart J..

